# May direct-to-consumer genetic testing have an impact on general practitioners’ daily practice? a cross-sectional study of patients’ intentions towards this approach

**DOI:** 10.1186/s12875-021-01428-6

**Published:** 2021-04-26

**Authors:** Christine Cohidon, Regula Cardinaux, Jacques Cornuz, Robin Chenal, Béatrice Desvergne, Idris Guessous, Daniela Cerqui, Daniel Widmer

**Affiliations:** 1 Center for Primary Care and Public Health (Unisanté), University of Lausanne, Lausanne, Switzerland; 2 Centre for Integrative Genomics (CIG), University of Lausanne, Lausanne, Switzerland; 3 Division and Department of Primary Care Medicine, Geneva University Hospitals, Geneva, Switzerland; 4 Institute of Social Sciences, Faculty of Social and Political Sciences, University of Lausanne, Lausanne, Switzerland

**Keywords:** DTCGP, General medicine’ Patients, Attitudes

## Abstract

**Background:**

Direct-to-consumer genetic testing (DTCGT) offers individuals access to information on their probable risks of suffering from a wide range of chronic diseases. General practitioners (GPs) will probably play a major role in supporting its use, but patients’ perception of DTCGT remain unclear. This study aimed to describe those attitudes and expectations and how they might affect GPs’ daily practices.

**Methods:**

In 2018–2019, a study related to the use of DTCGT for preventive care in general medicine was conducted among patients in Switzerland’s French-speaking areas. Data were collected in the waiting room using a self-administrated questionnaire about patients’ interest in DTCGT and what their attitudes might be if testing revealed an elevated risk of diabetes, colorectal cancer, or Alzheimer’s disease.

**Results:**

About 40% of the 929 participating (participation rate about 80%) patients had heard about DTCGT and, once the test had been explained, 43% reported that they would be interested in being tested. If that testing suggested an elevated risk of disease, the majority of patients reported that they would change their lifestyle (65%–81%, depending on the disease), request more examinations (63%–77%), and expect changes in their GP’s follow-up (48%–59%). Personal characteristics such as sex, age, urbanity, marital status, and perceived health were factors predictive of patients’ attitudes.

**Conclusion:**

Findings indicated that the generalization of DTCGT might affect GPs’ daily practices in terms of workload and knowledge about this approach. However, this result must be qualified by the fact that it is based on hypothetical situations.

**Supplementary Information:**

The online version contains supplementary material available at 10.1186/s12875-021-01428-6.

## Background

Personalized, precision, or genomic medicine based on DNA sequencing seems to offer good opportunities for improving overall public health. Initially developed for genetically-targeted cancer therapies, DNA sequencing can now also be applied to disease prevention (e.g., cancers, heart disease, and metabolic diseases) through the identification of patients’ risk profiles [1]. Although scientific and technological advances make this identification possible, it is questionable whether patients and health professionals are ready for the widespread introduction of this approach [2–4].

In recent years, direct-to-consumer genetic testing (DTCGT) has given individuals access to information on their risks of suffering from a wide range of chronic diseases [5–7]. With a few cells collected using an oral swab, patients can order a test online and receive their results by electronic mail or post. This practice is currently poorly regulated everywhere, and in Switzerland, DTCGT is used very little. The idea that DTCGT might be done out of curiosity or for fun seems potentially dangerous and clinically useless [8]. Furthermore, there is little knowledge about whether individuals are interested in the approach and how their results might lead them to change their health behaviors [9, 10].

Should DTCGT become widespread in the near future, general practitioners (GPs) will probably play a major role, both to accompany patients through the approach (interpretation of results) and on its potential clinical consequences [7, 11]. Their position at the heart of the health care system, their mission of prevention, and their privileged relationship with patients will quite naturally make them central in the approach’s development [12]. Several studies have emphasized that patients would expect their GP to help them with their results and manage the consequences [10, 13, 14].

In this context, extra demands on GPs might have several consequences on their practices. Those consequences could be related to having a minimum amount of knowledge about interpreting risk and the calculation of estimations, counselling patients and managing their fears and worries once results are known, and increasing workloads resulting from meeting patients new expectations and demands [2, 10, 15, 16]. The latter might include a desire for more consultations, examinations, and preventive counselling, as well as changes in how their diseases are managed [17–19].

The provision of general medicine is currently under strain in numerous countries due to too many patients, dissatisfaction with the work, and the perspective of a shortage of GPs [20, 21]. It is therefore particularly pertinent to anticipate the possible effects on general medicine due to the generalization of DTCGT.

The present study aimed first to describe patients’ knowledge and attitudes about DTCGT; second, it aimed to describe their intentions and expectations in case of elevated risk. Finally, this will help us to discuss how their attitude might affect GPs’ daily working practices.

As a preliminary step, we carried-out a qualitative exploratory study involving 10 patients. These individual interviews, conducted according to the comprehensive interview method [22], aimed at exploring the extent of patients' knowledge on the subject. The points underlined by the results were as follows: lack of knowledge of the procedure (confusion with patient-centered medicine), reluctance to undergo such tests (after explanations), major role of the GP in accompanying the approach, issue of costs and ethics.

## Methods

### Study design and population

In 2018, the Center for Primary Care and Public Health (Unisanté), in collaboration with Geneva University Hospitals’ (HUG) Division of Primary Care Medicine and the University of Lausanne’s Department of Social and Political Sciences launched a project on the use of DTCGT for preventive care in general medicine. The project’s aims were, firstly, to investigate patients’ perceptions, expectations and intentions with regard to DTCGT, and secondly, to discover GPs’ points of view, particularly concerning their current and future working practices. The project incorporated a phase examining two complementary, parallel groups—the patient group and GP group—. The present paper reports the results from the patient group.

This part of the study used the 277 randomly selected family physicians who were members of the Swiss Primary health care Active Monitoring (SPAM) network in 2015. This national network was created in 2012 from the comprehensive list of GPs created by combining the membership of the Association of Family Doctors and Pediatricians and the Swiss Society of General Internal Medicine. The physicians were asked whether they would be willing to participate in a research network. The representativeness of the network’s participants was cross-checked against national statistics on GPs’ sex, age, and rural or urban location, and it was considered satisfactory [23]. The present study only solicited the network’s French-speaking members (*n *= 84 GPs). Providing them with a random patient selection algorithm, we asked these GPs to invite their patients to participate in the study; when the selected patient refused to participate, participation was offered to the following one until 40 patients were recruited. No incentives were offered to either GPs or patients. Data collection took place between November 2018 and March 2019. Patients with cognitive problems or who were not fluent in French were excluded.

Because the questionnaire was anonymous, no health data were collected, and it was not possible to identify the participants from the data, the Human Research Ethics Committee of the Canton of Vaud (CER-VD), Switzerland, authorized the project under a simplified and accelerated procedure (Request number- 2018–00,160). This procedure does not request the written consent of patients (only oral consent). At the practice, patients received information about the study from the medical assistant and were free to refuse to participate.

### Data

Before beginning this study, we carried out a small exploratory qualitative study comprising interviews on the theme of DTCGT with five patients and five GPs. We used the results of this qualitative part to complete the list of topics from the existing literature that we wanted to study in patients. Thus we were able to develop a questionnaire adapted to our context [24] (see supplementary file). The questionnaire was anonymous. The patients, invited to participate by the medical assistant or the physician, filled it in the waiting room and put it directly in a dedicated box at the practice’s secretariat.

The questionnaire included a section on patients’ knowledge about DTCGT and interest in the approach and a section on what their attitudes might be if testing revealed an elevated risk of diabetes, colorectal cancer, or Alzheimer’s disease in comparison to the general population. These three specific diseases were chosen to explore patients’ attitudes to diseases with different degrees of severity and the possibilities of preventing them.

The questionnaire also collected classical sociodemographic and personal information namely gender, year of birth, education level, marital status, employment status and income. With regard to the present article’s aim to consider DTCGT’s potential impact on GPs’ daily working practices, we studied the following questions – both guide by existing literature and the qualitative exploratory phase-:

- Would you like to discuss DTCGT with your GP before deciding on whether to take this type of test? [25]
- When you receive your results, would you like to discuss them with a professional? If yes, with your GP? [10]
- Based on the test’s results (if testing revealed an elevated risk in comparison to the general population), do you think that [26–28]
  o you would change your lifestyle [18]?
  p you would like your GP to change your health management? [29]
  q you would ask your GP to perform complementary examinations (such as blood tests, colonoscopy, brain MRI, …) more frequently? [29]
- Do you think that you would like to be treated for this problem by a GP? [30]
- Knowing that you had an increased risk, do you think that you would be worried throughout your life? (Not at all worried / A little worried / Somewhat worried / Very worried / I don’t know) [31].

We tested the questionnaire (understanding and acceptability) with a dozen patients of different ages, genders and levels of education.

### Statistical analysis

We first made some descriptive statistical analyses to characterize patients’ knowledge of and attitudes to DTCGT. We described the changes they envisaged, lifestyle changes, and expected changes in health monitoring, should their tests predict an elevated risk of diabetes, colorectal cancer, or Alzheimer’s disease. We subsequently created three new variables describing the changes envisaged should a disease risk (any disease among diabetes, colorectal cancer, or Alzheimer’s disease) be higher than that of the general population. We performed bivariate and multivariate analyses to investigate the factors (independent sociodemographic and personal variables) predictive of these changes (dependent variables). We built three final multivariate models to study the variables of lifestyle change, changes in GPs’ disease management, and requests for more regular examinations. Statistical analyses were performed using STATA software (Version 14.2, Stata Corp, College Station, USA).

## Results

### Sample characteristics

Twenty-nine of 84 French-speaking GPs agreed to participate in the study (34% participation rate), and 929 of 1161 patients answered the questionnaire (~ 80% participation rate). Table 1 describes the participants, who were mostly men (57%), had a median age of 58 years old (18–100), and of whom 35% were retired and about 40% suffered from a chronic disease.

**Table 1:** Study population characteristics

Population	*n* (*N *= 929)	% or median
**Gender**	919	
Female	394	42.9
**Male**	525	57.1
**Age (median)**	916	58
**Area**	895	
Urban or peri-urban	681	**76.1**
**Rural**	214	**23.9**
**Marital status**	909	
Single	296	32.6
Couple	553	60.8
Other^a^	60	6.6
**Job status**	907	
Employed	415	45.8
Retired	313	34.5
Other	179	19.7
**Perceived health** (median /10^b^)	906	**7.5**
**Existing chronic disease**	834	
Yes	334	**40.5**
No	500	**59.5**
**Knowledge of DTCGT**	917	
Yes	366	39.9
**No**	**551**	**60.1**
**Interest in DTCGT to prevent disease**^c^	**911**	
Yes	395	43.4
Depending on the disease	157	17.2
I don’t know	146	16.0
No	213	23.4

^a^ Living with parents & do not want to answer; ^b^0 to 10, with 10 = very good perceived health; ^c^After explanations about the test

Forty percent of patients had previously heard about DTCGT. Once the DTCGT approach had been explained (via a short explanatory text in the questionnaire), a total of 43% of patients reported that they would be interested in taking a test and an additional 17% would do it only depending on the disease being tested for.

### General medicine patients’ knowledge of and attitudes to different aspects of direct-to-consumer genetic tests (Table 2)

**Table 2:** General medicine patients’ knowledge of and attitudes to different aspects of direct-to-consumer genetic tests (% of answers)

	Gender			Age				Area			Total
	Female	Male	*p*	< 50	50–65	> 65	*p*	Rural	Urban	*p*	
Would you like to discuss DTCGT with your GP before deciding on whether to take this type of test? (***n ***= 904^¥^)											
Yes	76.4	79.4	ns	80.1	76.6	77.9	ns	75.6	78.6	ns	78.1
No	15.8	15.1		12.9	18.1	15.1		17.4	14.9		
Do not know	7.8	5.5		6.9	5.3	7.0		7.0	6.5		
Who should receive the test results? (***n ***= 925^a^)											
My GP exclusively or both me and my GP	92.9	92.9	ns	92.2	92.5	92.1	ns	92.4	93.1	ns	92.9
Only myself	5.9	3.2		6.0	4.2	2.8		4.2	4.2		
Other	1.3	3.8		2.8	3.2	2.1		3.3	2.7		
How worried would you be while waiting for the test results? (***n ***= 922^a^)											
Somewhat or very worried	20.7	31.3	***	35.6	28.1	15.0	***	27.2	26.8	***	26.7
Not at all worried or a little worried (or do not know)	79.3	68.7		64.3	71.9	85.0		72.8	73.2		
Knowing that you had an increased risk, do you think that you would be worried throughout your life? (***n ***= 898^a^)											
Somewhat or very worried	26.5	34.8	**	37.9	33.4	21.1	10^–3^	34.4	29.7	ns	31.5
Not at all worried or a little worried (or do not know)	73.5	65.2		62.1	66.6	78.9		65.6	70.3		
When you received your results, would you like to discuss them with your GP? (***n ***= 929^a^)											
Yes	90.9	92.4	ns	91.2	91.6	92.8	ns	87.8	93.5	**	91.7
No	9.1	7.6		8.8	8.4	7.2		12.2	6.5		
Do you think you would like to be treated for this problem by a GP? (***n ***= 929^a^)											
At least by a GP	71.3	70.3	ns	69.1	75.0	68.7	ns	72.4	71.2	ns	70.7
By another professional	28.7	29.7		30.9	25.0	31.3		27.6	28.8		

^*^ 5.10^–2^ ≤ *p *< 10^–2^; ** 10^–2^ ≤ *p *≤ 10^–3^; *** *p *≤ 10^–3^; *ns* Not significant; ^a^ total respondents to the question

The vast majority of patients would want to discuss DTCGT with their GP before taking a test. Once the test would be done, 93% thought that their GP at least should receive the results (among them 17% thought that only their GP (not even themselves) should receive the results) and more than 90% would want to discuss those results with them. In addition, almost 80% of women said they would not worry while waiting for their test results (vs 69% of men) and 85% of participants aged >  = 65 years old (vs 64% in < 50 years old participants) reported that they were not worried, thus revealing significant differences for sex and age. In results showing a high risk of disease, about one-third of men and one-third of patients < 50 years old thought they would remain worried throughout their lives (revealing statistically significant differences for sex and age).

### Consequences to patient lifestyles of direct-to-consumer genetic tests reporting elevated disease risks in comparison to the general population

Most patients (59%) reported that they would change their lifestyle (regarding food, physical activity, alcohol consumption, or smoking habits) if DTCGT reported them as belonging to a high risk category in comparison to the general population, whatever the disease. Additionally, 50% would request further complementary examinations. Lastly, patients would want their GP to change their health management (e.g., in terms of consultation frequency) should the test report an elevated risk of disease. Whatever the disease, women would be less likely to change their health behaviors than men (OR: 0.68 [0.52–0.90]). Patients living in urban areas would request more changes in their health management, with regards to their GP’s working practices and to complementary examinations, with ORs of 1.53 [1.08–2.16] and 1.37 [0.99–1.88], respectively. Younger patients (< 50 years old) would request more complementary examinations than older ones (OR = 1.78 [1.27–2.50]). However, patients living with their parents less frequently reported the intention to change their lifestyle (OR = 0.40 [0.23–0.72]) or the desire to change their health management (OR = 0.42 [0.21–0.83]) (Tables 3 and 4). In the final multivariate models, neither level of education nor existing chronic diseases were associated with patients’ attitudes.

**Table 3:** Consequences of the results of direct-to-consumer genetic tests on patients’ health behaviors, according to disease (% of patients answering “yes” to the questions)

	N	Gender			Age				Area			**Total**
		Female	Male	*p*	< 50	50–64	> = 65	*p*	Rural	Urban	*p*	
**Based on the test’s results, do you think that you would change your lifestyle?**												
Diabetes	901	77.3	84.2	**	85.3	82.8	75.5	**	77.8	82.5	ns	**81.1**
Colorectal cancer	879	78.6	82.3	ns	81.3	81.5	79.5	ns	79.9	81.2	ns	**80.7**
Alzheimer’s disease	876	60.4	69.0	**	63.1	66.9	65.8	ns	59.8	67.0	ns	**65.2**
Systematically, whatever the disease	882	54.0	62.5	*	57.1	62.3	57.2	ns	54.4	60.2	ns	**58.7**
**Based on the test’s results, do you think you would like your GP to change your health management?**												
Diabetes	868	46.1	49.3	ns	44.6	48.1	51.9	ns	43.3	49.1	ns	**47.8**
Colorectal cancer	851	59.4	59.2	ns	61.2	58.1	57.3	ns	62.4	58.6	ns	**59.1**
Alzheimer’s disease	867	53.9	57.8	ns	49.7	59.4	59.6	*	51.2	57.2	ns	**55.9**
Systematically, whatever the disease	860	35.2	36.4	ns	34.5	38	34.4	ns	29.1	38.2	*	**35.8**
**Based on the test’s results, do you think that you would ask your GP to perform complementary examinations more frequently?**												
Diabetes	896	67.4	68.4	ns	73.7	67.1	61.8	**	68.3	67.8	ns	**67.4**
Colorectal cancer	892	75.5	78.9	ns	81.6	78.6	70.9	**	76.8	77.5	ns	**77.1**
Alzheimer’s disease	874	60.7	66.0	ns	67.2	63.9	58.4	**	55.9	65.7	**	**63.2**
Systematically, whatever the disease	878	49.1	51.3	ns	56.3	50.8	42.1	**	44.4	52.2	*	**49.9**

^*^ 5.10^–2^ ≤ *p *< 10^–2^; ** 10^–2^ ≤ *p *≤ 10^–3^; *** *p *≤ 10^–3^; ns: not significant

**Table 4:** Factors associated with changes in health behavior if testing revealed an elevated risk of disease in comparison to the general population (whatever the disease), using logistic regression

	Lifestyle changes N* = 860		Desire for a change in the GP’s health management N* = 820		Complementary examinations requested N* = 845	
	OR and 95% CI*		OR and 95% CI*		OR and 95% CI*	
	Bivariate	Multivariate	Bivariate	Multivariate	Bivariate	Multivariate
**Gender:** Female	0.70 [0.53–0.92]	**0.68** **[0.52–0.90]**	0.95 [0.72–1.26]	**-**	0.91 [0.70–1.19]	**-**
**Age:** Ref. > = 65	1	**-**	1	**-**	1	**1**
50–64	1.23 [0.89–1.73]		1.19 [0.84–1.69]		1.42 [1.01–1.99]	**1.43** **[1.01–2.01]**
< 50	0.99 [0.71–1.38]		1.00 [0.71–1.43]		1.77 [1.27–2.47]	**1.78** **[1.27–2.50]**
**Urban or peri-urban area**	1.27 [0.93–1.74]	**-**	1.51 [1.09–2.12]	**1.53** **[1.08–2.16]**	1.37 [1.00–1.87]	1.37 [0.99–1.88]
**Marital status:** Ref. Single	1	1	1	1	1	
Couple	1.13 [0.84–1.52]	1.15 [0.85–1.55]	0.93 [0.69–1.27]	0.98 [0.72–1.35]	0.96 [0.72–1.29]	
With parents	0.41 [0.23–0.73]	**0.40** **[0.23–0.72]**	0.40 [0.20–0.79]	**0.42** **[0.21–0.83]**	0.99 [0.56–1.74]	
**Employment:** Ref. Retired	1	**-**	1	**-**	1	-
Employed	1.24 [0.91–1.69]		1.04 [0.75–1.44]		1.39 [1.02–1.88]	
Other	0.75 [0.51–1.09]		1.18 [0.79–1.75]		1.42 [0.97–2.08]	
**Level of education:** Ref. No qualification	1	**-**	1	**-**	1	-
Upper secondary	1.07 [0.75–1.60]		0.90 [0.60–1.36]		0.89 [0.60–1.33]	
Post-secondary	1.47 [0.98–2.21]		0.79 [0.52–1.21]		1.06 [0.70–1.58]	
**Existing chronic disease**	0.16 [0.86–1.55]	**-**	1.36 [1.01–1.84]	**-**	1.21 [0.91–1.60]	-
**Perceived health** (from 0 (bad) to 10 (good))	1.05 [0.96–1.14]	**-**	0.92 [0.85–1.01]	0.93 [0.85–1.01]	0.96 [0.88–1.04]	

*OR* Odds ratio, *95% CI* Confidence interval, N* Final model

These changes varied according to the potential disease (Table 3). The reported intentions to change lifestyle and to use more health care services were more prevalent for diabetes and colorectal cancer than for Alzheimer’s disease.

## Discussion

DTCGT is as yet not a generalized approach in Switzerland. However, our results showed that more than four in ten general medicine patients would be interested in this kind of testing. The patients surveyed stated that their GP should be at the heart of the process, including discussions with them before testing and once the results have been received. The study also showed that genetic testing might affect GPs’ daily working practices through -changes to patients’ behaviors or attitudes, with differences depending on the disease tested for: the willingness to change their lifestyle, the health management they would expect from their GP in terms of the frequency of consultation, and the desire to have more frequent complementary examinations. These changes varied according to sociodemographic and personal characteristics such as sex, age, urbanity, marital status, and perceived health.

The sample’s patients put their GP at the heart of any DTCGT approach, wishing to involve them before testing, on receipt of the results, and in the management of the disease predicted to have an elevated risk. These results could be because the survey was conducted in GPs’ waiting rooms. However, the literature shows inconsistent findings about patients’ disclosure about test results and their intent to discuss those results with their GP. Several American studies conducted in the general population have reported that people did not want to share or did not share their DTCGT results with a physician [8, 13, 32]. For instance, Kaufman’s general population study of people who had done DTCGT showed that only 20% had reported that they had discussed their results with their GP (28% had spoken with a health care professional) [13]. Wasson et al.’s qualitative study reported that less than half of their participants expressed an intention to discuss DTCGT or its results with their GP [10].More recently, McGrath reported an even lower percentage (11%) in the same type of population [32]. In contrast, in populations of primary care patients, studies have shown greater use of GPs to help understand and decide what to do with testing results, but again, with mixed findings [10, 14, 27]. On the physicians’ side, American GPs seem to be prepared to discuss DTCGT results with their patients to provide them with an accurate interpretation [2, 19, 33]. In any event, many authors agree on the need to discuss results with a professional as even though they may be easy to understand, they can often be misinterpreted [12]. The fact that GPs should be the professional with whom DTCGT results are discussed implies that they should be knowledgeable about the approach. Although discussing medical uncertainty should not be challenging to GPs, they would benefit from additional training about how DTCGT results are produced, how to interpret them, and their limitations [11, 34–36].

Our results also indicated that generalized genetic testing might affect GPs’ daily workloads. Firstly, GPs might be solicited more frequently by their patients seeking help to change their lifestyle and health behaviors if they receive results indicating a high risk of developing a disease. GPs generally perceive the provision of preventive care to be one of their primary missions. However, in many countries, these activities are often difficult to implement because GPs lack time, knowledge, and confidence in that provision having any effect; furthermore, in some countries, providing preventive care is not highly valued [37, 38]. In the part of our study looking at GPs’ opinions of and attitudes to DTCGT, they seemed to consider genetic testing to be just another tool (accepted for publication [39]). It should also be noted that the literature reveals that patients’ intentions to adopt healthier behaviors following DTCGT are not always matched by real changes in their health behavior [14, 17–19]. In contrast, as a perverse effect of the approach, patients categorized as lower risk may be tempted to be more relaxed with their health behaviors even though the risk for certain diseases may remain high in absolute value in the general population. Additionally, this could be especially deleterious since levels of risk are provided in comparison to the general population and not as absolute risk.

Secondly, the vast majority of patients reported that if presented with a higher risk of developing a chronic disease, they would want their GPs to change their health care management. Particularly, this might imply more consultations. Considering GPs’ contemporary heavy workloads and the predicted shortage of them in many Western countries, this would appear to be problematic. In addition, patients with an elevated risk of a disease might request additional examinations, also leading to an increase in health care costs. Additionally in this context, incidental findings should also be managed by the physicians.

One potential solution to the extra burden which DTCGT might put on GPs is the presence of other specialized professionals dedicated to this issue at the consultancy. These professionals would have to be trained to answer patients’ questions.

Our results indicate that patients’ expectations after DTCGT and their attitudes once they have received their results can vary according to sociodemographic factors. For instance, patients living in urban areas may be more likely to expect changes in their health management than patients in rural areas, as may younger patients versus older ones (except for the very young who still live with their parents). Men were more likely than women to report their intention to change their lifestyle. Actually, differences between gender are observed for almost all issues, whether they relate to opinions or intentions to change behavior (sometimes not statistically significant). Outside of this context, it is known that women generally adopt healthier lifestyles than men [40]. Hence, major changes are likely to be more difficult to implement for women. In addition, it can be hypothesized that a worrisome outcome may be more of a driver for change in men. The literature seems to offer inconsistent results regarding this issue [31, 41]. Other sociodemographic differences regarding health attitudes and behaviors are typical and have already been described in relation to DTCGT, especially for age, and urbanity [9, 13, 27, 42, 43]. Patients’ attitudes may also vary according to the disease. A few studies have already investigated this point, and it could be a crucial one. Patients’ current attitudes seem to differ according to the severity of the disease and the potential to prevent and treat it. On this point, patients’ attitudes may well be close to those of their physicians, as the latter perceive screening tests to be most useful if there are treatment measures which can be applied to the disease discovered [34].

According to our results, only a small proportion (a quarter to one third) of patients reported that they would be worried either while waiting for their results or for the rest of their lives if they were at a high risk of disease. This concern was more likely to affect younger people and men. However, physicians often report the need to manage patients’ anxiety about test results [16, 34]. This discrepancy between patients’ and physicians’ attitudes may have resulted from the fact that patients were responding to a questionnaire about a hypothetical situation, whereas physicians live those experiences.

Finally, our study aimed to better grasp how patients’ expectations with respect to DTCGT might affect GP daily practice. Thus, this study did not discuss the proper limitations of the DTCGT. The related questions are however of great importance. The first one concerns the reliability of current tests available online. Albeit recent regulatory actions by FDA have certainly improved the overall testing quality, false positive seems to still be not so rare [1]. The second one is the clinical validity and utility of these tests. There is indeed a high risk of misinterpreting the so-called low risk and high risk value. The odd ratios of having a given disease are obtained comparing the two tails of a distribution, ignoring the vast proportion of individual falling between the two tails of this distribution. This leads to a major overestimation of the clinical value of the tests, particularly in the case of polygenic risk scores [44, 45]. Considering the previous discussion on how these tests might affect primary care, for both the patients and the GPs, this major point still needs to be addressed.

### Limitations

One major limitation to estimating the impact of DTCGT on GPs’ daily working practices is that the data collected are attitudes-related and based on hypothetical situations, not real behaviors. Furthermore, studies examining opinions about and attitudes to DTCGT do not take into account the reliability of the results. Some professionals raise concerns about the poor performance of the predictions, such as their low predictive value and the common existence of false positive and false negatives [1]; others underline limitations in the calculation of the probabilities [46]. The present study was conducted in GPs’ waiting rooms, which may have introduced biases in patient selection (e.g., because of lack of time or lack of interest), despite the algorithm provided, and in patients’ answers about their GP’s involvement in the approach. The patients participation rate was high. However, this figure was based on limited data, as less than 50% of physicians reported this information.

## Conclusion

The generalization of direct-to-consumer genetic testing (DTCGT) might have an impact on GPs’ daily practices. Primary care professionals and primary health care systems should prepare for this. The most obvious first step in this direction is increased training for GPs on the subject of personalized medicine. Providing GPs with regular information on the evolution of genetic testing also seems necessary. Finally, clear guidelines from professional medical associations could also be a good option. This is the strategy that has been adopted in the UK, where policy makers or scholarly societies have recommended, for instance, that GPs do not provide genetic testing without a relevant clinical indication [47].

## Supplementary Information

**Additional file 1.** Supplementary file1

## Data Availability

The datasets used and/or analysed during the current study are available from the corresponding author on reasonable request. Because the questionnaire was anonymous, no health data were collected, and it was not possible to identify the participants from the data, the Human Research Ethics Committee of the Canton of Vaud (CER-VD), Switzerland, authorized the project under a simplified and accelerated procedure (Request number- 2018–00160). This procedure does not request the written consent of patients (only oral consent). At the practice, patients received information about the study from the medical assistant and were free to refuse to participate.

## Abbreviations

DTCGT: Direct-to-consumer genetic testing
GP: General practitioner
HUG: Geneva University Hospitals
SPAM: Swiss Primary health care Active Monitoring
OR: Odd ratio
