# Supplementary material for: May direct-to-consumer genetic testing have an impact on general practitioners’ daily practice? a cross-sectional study of patients’ intentions towards this approach
Source: BMC Fam Pract. 2021 Apr 26;22:79. doi: 10.1186/s12875-021-01428-6 (PMC8077756; doi:10.1186/s12875-021-01428-6)
Supplement: Supplementary file 1 — Additional file 1. Supplementary file1 [file 12875_2021_1428_MOESM1_ESM.pdf]

PATIENTS' EXPECTATIONS AND OPINIONS ON THE BENEFITS OF PERSONALISED MEDICINE FOR THE PREVENTION OF CHRONIC ILLNESSES IN GENERAL MEDICINE

Please read the following box carefully:

In the not too distant future, your doctor could be able to offer you a test to determine your genetic risks of developing certain diseases, such as cancers, heart diseases, hypertension, diabetes, obesity, Alzheimer's disease and inflammatory diseases. All this would require would be for you to rub the inside of your cheek with a cotton swab in order to gather a few cells and then send it to a laboratory. Testing your cells would give you an estimation of your risk of developing a particular disease during your lifetime in comparison to the average risk facing the general population. For example, you might be given a 1-in-3 chance of having a heart attack, in comparison to the 1-in-5 chance faced by the general population.

1. Have you ever heard about the possibility of this type of test before?

☐ Yes ☐ No ☐ I don't know

Suppose that you decide to take the test…

2. How worried would you be while awaiting your test results?

☐ Not at all worried ☐ A little worried ☐ Quite worried ☐ Very worried ☐ I don't know

3. Who do you think should receive the test results?

☐ You alone ☐ Your doctor alone ☐ Both at the same time ☐ Somebody else ....

4. Do you think that you would be able to understand your test results without the aid of a professional?

☐ Yes ☐ No ☐ I don't know

5. On receiving your results, would you like to discuss them with a professional?

☐ Yes ☐ No ☐ I don't know

If *Yes*, with what kind of professional?

(more than one answer is possible) ☐ Your general practitioner ☐ A geneticist ☐ A nurse trained in the field ☐ Another professional ....

Scenario 1. Suppose that the test result indicates that you have a higher risk of developing diabetes than the general population does.

**6. Do you think that you would want…**	Yes	No	Don't know
a. to change your lifestyle (diet, sport, tobacco, alcohol, etc.)?			
b. the monitoring by your family doctor to change (e.g. more consultations)?			
c. to undergo complementary examinations more regularly (blood tests, etc.)?			

d. Who do you think you would want to take care of this problem?

(More than one answer is possible) ☐ Your general practitioner ☐ A diabetes specialist ☐ A geneticist ☐ A nurse trained in the field ☐ Another professional

Scenario 2. Suppose that the test results indicate that you have a greater risk of developing colon cancer than the general population does.

**7. Do you think that you would want…**	Yes	No	Don't know
a. to change your lifestyle (diet, sport, alcohol, etc.)?			
b. the monitoring by your family doctor to change (e.g. more consultations)?			
c. to undergo complementary examinations more regularly (blood test, hemoccult test, colonoscopy, etc.)?			

d. Who do you think you would want to take care of this problem?

(More than one answer is possible) ☐ Your general practitioner ☐ A cancer specialist ☐ A geneticist ☐ A nurse trained in the field ☐ Another professional

Scenario 3. Suppose that the test results indicate that you have a greater risk of developing Alzheimer's disease than the general population does.

**8. Do you think that you would want…**	Yes	No	Don't know
a. to change your lifestyle (diet, sport, crosswords, etc.)?			
b. the monitoring by your family doctor to change (e.g. more consultations)?			
c. to undergo complementary examinations more regularly (blood tests, brain MRI, etc.)?			

d. Who do you think you would want to take care of this problem?

(More than one answer is possible) ☐ Your general practitioner ☐ A neurologist ☐ A geneticist ☐ A nurse trained in the field ☐ Another professional

In general, after having read about these examples…

9. Would you like to take this test in order to get an estimation of your risks of developing different diseases throughout the course of your life?

☐ Yes ☐ No ☐ I don't know ☐ It depends on the illness

10. Whatever your reply, would you change your mind…

a. if the test's costs were covered by your health assurance?

☐ Yes ☐ No ☐ I don't know

b. if you had to pay for the test out of your own pocket (around CHF 300)?

☐ Yes ☐ No ☐ I don't know

11. Would you like your loved ones to take this test in order to get an estimation of their risks of developing different diseases throughout the course of their life?

a. Your parents and/or brothers and sisters

☐ Yes ☐ No ☐ I don't know ☐ Plus de parents

b. Your spouse/partner

☐ Yes ☐ No ☐ I don't know ☐ I have no spouse/partner

c. Your children

☐ Yes ☐ No ☐ I don't know ☐ I have no children

12. Would you like to be able to talk to your general practitioner before deciding on whether to take this kind of test?

☐ Yes ☐ No ☐ I don't know

13. In general, do you think that you might be worried throughout your life if you knew that those risks were greater?

☐ Not at all worried ☐ A little worried ☐ Quite worried ☐ Very worried ☐ I don't know

14. Would you agree to your results being shared with…

a. your loved ones? ☐ Yes ☐ No ☐ I don't know

b. medical researchers? ☐ Yes ☐ No ☐ I don't know

c. your health assurance company? ☐ Yes ☐ No ☐ I don't know

15. Do you suffer from any chronic illnesses? ☐ Yes ☐ No ☐ I don't know

If yes, ☐ Hypertension ☐ Diabetes ☐ Cholesterol ☐ Cancer ☐ Heart disease ☐ Depression ☐ A genetic illness; please, state which one: …………………………………….

16. Does anybody in your close family (parents, brother or sister, spouse or partner, children) suffer from a genetic disease (e.g. Down's syndrome, muscular dystrophy, …)?

☐ Yes ☐ No ☐ I don't know

17. How would you rate your overall health status?

Poor /---/---/---/---/---/---/---/---/---/---/ Excellent

0---1---2---3---4--5---6--7---8--9--10

Please put a cross somewhere along the scale between 0 and 10 to situate your overall state of health, with 10 being the best health status possible and 0 being the worst.

PERSONAL DATA

18. Sex ☐ Male ☐ Female

19. What was your year of birth? ....

20. What is your postal code? ....

21. Who do you live with?

☐ Alone ☐ Alone with children ☐ In a couple with children ☐ In a couple without children ☐ With my parents ☐ I don't want to answer

22. What is your educational level?

☐ Did not finish compulsory schooling ☐ Finished compulsory schooling ☐ General vocational training (apprenticeship) ☐ Higher education

23. What is your employment status?

☐ Apprentice ☐ Student ☐ Employee ☐ Retired ☐ Unemployed ☐ Other non-employed statuses, housewife/husband, receiving disability benefits, …

24. What is your household's approximate net monthly income?

☐ < CHF 4,500 ☐ CHF 4,500–6,000 ☐ CHF 6,000–9,000 ☐ > CHF 9,000

☐ I don't wish to reply

THANK YOU FOR YOUR PARTICIPATION!
